# Morbidity and patient characteristics on acute presentation with sore throat: a multicentre national audit

**DOI:** 10.1308/rcsann.2025.0059

**Published:** 2026-01-20

**Authors:** T Ton, A Sheldon, N Duncan, R Gohil, K Stewart, P Sooby, R Sproat, R Hurley, K To, VV Wilmont, L McMurran, S Hey, CM Moen, S Corson, L Clark, CM Douglas

**Affiliations:** ^1^ NHS Greater Glasgow and Clyde, UK; ^2^ University of Glasgow Medical School, UK; ^3^ NHS Lothian, UK; ^4^ NHS Tayside, UK; ^5^ NHS Ayrshire and Arran, UK; ^6^ NHS Grampian, UK; ^7^ NHS Highland, UK; ^8^ University of Strathclyde, UK

**Keywords:** Tonsillitis, Pharyngitis, Tonsillectomy, Surgery, Otolaryngology

## Abstract

**Introduction:**

Sore throat is one of the most common reasons for an acute ear, nose and throat (ENT) admission. Recurrent tonsillitis can be treated definitively by tonsillectomy, but patients must fulfil Scottish Intercollegiate Guideline Network (SIGN) guidelines to be eligible. The aim of this audit was to assess the throat morbidity of patients admitted with ‘sore throat’ to ENT wards across Scotland.

**Methods:**

A multicentre prospective audit was conducted across six Scottish ENT units over 4 months to assess demographics, risk factors and episode history in patients admitted with sore throat.

**Results:**

Some 279 patients were included: 63.9% were for admitted for tonsillitis, 35.7% for quinsy and 0.4% for deep neck infection. The mean age was 30.1 years (range 6–73 years). Most had reported 0–1 episodes of tonsillitis in the previous 4 years (58.5%–76.6%), with 41.3%–66.2% reporting no antibiotic treatment for sore throats in that time. Prior to admission, 48.7% had been prescribed antibiotics by a general practitioner (GP), and 16.1% had a history of hospital admission for tonsillitis. Only 25.6% of tonsillitis admissions met SIGN tonsillectomy criteria.

**Conclusions:**

Most patients admitted with sore throat in Scotland had low numbers of previous throat complaints. Fewer than half had received antibiotics from a GP before admission. One-quarter met SIGN criteria for tonsillectomy.

## Introduction

The symptom of sore throat is a common condition that is caused by a viral or bacterial infection. Common conditions that cause sore throat include tonsillitis, pharyngitis and glandular fever. These conditions are normally self-limiting and treated conservatively in the community setting; however, patients may be admitted to secondary care if they require intravenous therapy (whether this be fluids, steroids or antibiotics) and/or surgical intervention. Hot tonsillectomy is not common practice in the Scotland; however, a recent systematic review has demonstrated that quinsy tonsillectomy is safe, provides instant relief of symptoms and is not significantly associated with higher postoperative haemorrhage than interval tonsillectomy.^1^ The spike in deaths associated with invasive Group A streptococcus disease in recent years highlights the risk to life from a sore throat.^2^

In 1999, the Scottish Intercollegiate Guideline Network (SIGN) guidelines for sore throat and indications for tonsillectomy (Guideline 117) were published because of the significant burden of sore throat on health service resources in the United Kingdom (UK) – the guideline was further updated in 2005 and revised in 2010.^3^ The guidance outlines the general management of sore throat including the use of antibiotics and the criteria for surgery in the form of tonsillectomy. The widely accepted episodes of tonsillitis eligible for referral for surgery are as follows: sore throats that are due to acute tonsillitis, in which the episodes are disabling and prevent normal function; seven or more well-documented, clinically significant, adequately treated episodes of tonsillitis in the preceding year; five episodes in each of the preceding 2 years; or three episodes in each of the preceding 3 years. However, the original SIGN criteria were based on the 1984 Paradise paper that investigated the role of tonsillectomy in severely affected children, which equated to the ‘7,5,3’ threshold.^4,5^ Furthermore, the precursor 1976 Paradise and Bluestone paper, stated “for investigational purposes, the criteria we apply are necessarily arbitrary. Hence, they are not meant to serve as guidelines for physicians in practice”. Extrapolation of the evidence to an adult population for the SIGN guidelines and the lack of science in the original papers has not stopped the significant adherence to SIGN guidelines that we see today, despite the lack of evidence for the ‘7,5,3’ rule.

Since introduction of the guideline, there has been a 48% decrease in the number of tonsillectomies performed in Scotland, with rates remaining relatively steady over the past few years. Over the same period, rates of hospital admissions for sore throat and tonsillitis have increased: tonsillitis by 143%, peritonsillar abscess (quinsy) by 116% and deep neck space infections (DNSI) by 306%.^6^ The Scottish Reduction in Antibiotic Prescribing policy was further introduced in 2012, reducing the number of antibiotics prescribed in primary care.^7^ Prescriptions for antibiotics in hospitals have increased in the same period.^8^ Furthermore, the virulence of the Group A streptococcal organism, the main causative bacterial organism of tonsillitis, is increasing, as is its resistance to macrolide antibiotics.^9,10^

The aim of this national multicentre prospective audit was to obtain patient-level preadmission throat morbidity data on a cohort of patients admitted to secondary care pre-COVID.

## Methods

### Design and implementation

The audit was a national multicentre prospective study to establish the current status of a patient's tonsil morbidity prior to admission to an ear, nose and throat (ENT) ward with ‘sore throat’. Data were collected across Scotland at six ENT sites over a 4-month period, before the COVID pandemic (2017) using a paper pro-forma (Appendix 1 – available online). The six sites involved in the audit were: Queen Elizabeth University Hospital, Glasgow; Crosshouse Hospital, Kilmarnock; St Johns Hospital, Livingston; Ninewells Hospital, Dundee; Queen Victoria Hospital, Kirkcaldy; and Raigmore Hospital, Inverness.

### Ethics

Research ethics committee advice was sought using the National Health Service (NHS) Health and Research Authority and Medical Research Council website and was deemed not required because this was a clinical audit.

### Data collection

The inclusion criteria were any patient admitted to a Scottish ENT inpatient ward with a condition causing the symptom of ‘sore throat’, including tonsillitis, peritonsillar abscess or DNSI. Patients admitted with throat pain secondary to malignancy were excluded. Each site involved in the audit was assigned an ‘audit lead’ who oversaw the data collection of this audit. Data were collected using anonymous paper questionnaires, which were then uploaded to a secure server and transferred to an electronic database using double-data entry methods.

The data collected included patient demographics, such as age, postcode, sex and smoking status. Postcode information was used to identify the Scottish Index of Multiple Deprivation (SIMD) 2016 quintile for each locale. SIMD quintiles were obtained using a look-up table obtained from the Scottish government website (http://www.gov.scot/Topics/Statistics/SIMD).

Tonsil morbidity data were collected including: number of events of sore throat/tonsilitis in the preceding 4 years; how many events of sore throat/tonsilitis were reported to the patient's general practitioner (GP); how many events of sore throat/tonsilitis were treated with antibiotics; whether antibiotics had been commenced prior to the current hospital admission; and whether the patient had previously discussed surgical intervention for sore throat prior to the current admission. Diagnosis of admission and whether the patient met the criteria for tonsillectomy from SIGN Guideline 117 was also recorded.

### Statistical analysis

Categorical variables were summarised using number and percentage, numerical variables were summarised using mean and range. Statistical modelling techniques, such as logistic regression, ordinal logistic regression and general linear modelling, were used to see whether the outcome measures (number of events, number reported to GP, number treated with antibiotics, diagnosis) were associated with the demographics (age, SIMD, sex, smoking status). Statistical modelling techniques were used to assess associations using a univariate analysis with a 5% significance level.

## Results

### Demographics of the study participants

In total, 279 participants were recruited to the study; of whom 162 (58.1%) were from Queen Elizabeth University Hospital (Table 1). There were 152 female (54.5%) respondents to the survey and 114 male (40.9%) respondents; 13 (4.7%) replied ‘prefer not to say’ (Table 1).

**Table 1:** Demographics for study participants

Variable	Number (%)
Hospital	
Crosshouse	31 (11.1)
St Johns	32 (11.5)
Ninewells	18 (6.5)
Queen Victoria	17 (6.1)
Queen Elizabeth	162 (58.1)
Raigmore	19 (6.8)
Sex	
Female	152 (54.5)
Male	114 (40.9)
Prefer not to say	13 (4.7)
Smoking status	
Smoker	50 (19.8)
Non-smoker	172 (68.3)
Ex-smoker	30 (11.9)
Scottish Index of Multiple Deprivation quintile	
1	83 (31.4)
2	57 (21.6)
3	40 (15.2)
4	43 (16.3)
5	41 (15.5)

Of the 279 study participants, 252 (90.3%) provided information on their smoking status. Of these, 50 (19.8%) said they were smokers, 172 (68.3%) said they were non-smokers, and 30 (11.9%) said they were ex-smokers (Table 1).

SIMD quintiles were calculated for 264 (94.6%) of the 279 study participants. Reasons for missing information were an incomplete postcode or a postcode not recognised by the SIMD scot.gov look-up table, which may indicate a new postcode. Of the 264 study participants with postcode information, 83 (31.4%) had a SIMD quintile of 1, indicating that they came from the most deprived areas, and 57 (21.6%) had a SIMD quintile of 2. Similar numbers came from areas with SIMD quintiles of 3, 4 and 5 (Table 1).

The mean age for the 279 survey responders was 30.1 years (range 6–73 years). Mean age was highest for St Johns Hospital (34.2 years) and lowest for Ninewells Hospital (27.4 years). Three of the hospitals had mean ages of less than 30 years (Ninewells, 27.4 years; Queen Victoria, 27.6 years; and Queen Elizabeth, 29.8 years) (Table 2).

**Table 2:** Mean age and age range (years) for study participants

Variable	Mean	Range
Age (years)		
All respondents	30.1	6–73
Crosshouse	30.9	6–67
St Johns	34.2	17–72
Ninewells	27.4	15–49
Queen Victoria	27.6	9–47
Queen Elizabeth	29.8	15–73
Raigmore	30.0	14–62

### Number of sore throat events

Categorical groupings were used to collect information on the number of tonsillitis/sore throat events for the years 2013–2014, 2014–2015, 2015–2016 and 2016–2017. Here, participants could select between 0–1, 2–4, 5–6 and 7 or more.

Over the four periods of the study, the overwhelming majority of patients had reported 0–1 episodes of tonsillitis (*n* = 159–209, 58.5%–76.6%) before admission. The least number of patients reported 7 or more episodes (*n* = 4–7, 2.6%–4.8%). Table 3 summarises the number of events over each year.

**Table 3:** Number of sore tonsillitis events each year

	Number (%)
2013–2014	
0–1	209 (76.6)
2–4	42 (15.4)
5–6	18 (6.6)
7 or more	4 (1.5)
2014–2015	
0–1	198 (73.1)
2–4	47 (17.3)
5–6	19 (7.0)
7 or more	7 (2.6)
2015–2016	
0–1	182 (67.2)
2–4	55 (20.3)
5–6	27 (10.0)
7 or more	7 (2.6)
2016–2017	
0–1	159 (58.5)
2–4	74 (27.2)
5–6	26 (9.6)
7 or more	13 (4.8)

Ordinal logistic regression modelling showed that there was evidence of an association with age (*p* = 0.042), and no evidence of an association with smoking status (*p* = 0.142), sex (*p* = 0.226) and SIMD (*p* = 0.280). Modelling suggests that an increase in age (years) was associated with a decrease in the odds of having more tonsillitis events (odds ratio [OR] 0.98, 95% confidence interval [CI] 0.96–1.00).

### Events of sore throat reported to a GP

Over the 4-year period, the overall majority of patients reported that ‘none’ (40.4%–63.5%) of their reported events of sore throat were reported to a GP. A summary of these results is given in Table 4.

**Table 4:** Number of reported sore throat events to the general practitioner

	Number (%)
2013–2014	
All	41 (15.0)
Almost all	22 (8.0)
Half	20 (7.3)
Less than half	17 (6.2)
None	174 (63.5)
2014–2015	
All	41 (15.1)
Almost all	24 (8.8)
Half	15 (5.5)
Less than half	20 (7.4)
None	172 (63.2)
2015–2016	
All	45 (16.6)
Almost all	25 (9.2)
Half	18 (6.6)
Less than half	25 (9.2)
None	158 (58.3)
2016–2017	
All	80 (29.4)
Almost all	33 (12.1)
Half	32 (11.8)
Less than half	17 (6.3)
None	110 (40.4)

Ordinal logistic regression showed that there was no evidence of an association between SIMD (*p* = 0.232) and smoking status (*p* = 0.332), but there was evidence of an association with age (*p* = 0.022) and sex (*p* = 0.01).

For age, modelling suggests that an increase in age (years) was associated with increased odds of not reporting events to a GP (OR 1.02, 95% CI 1.00–1.04).

For sex, modelling suggests that the odds of males not reporting events to a GP was less than that for respondents who replied preferred not to say (OR 0.59, 95% CI 0.18–1.93). Similarly, the odds of females not reporting events to a GP was less than that for those who preferred not to say (OR 0.277, 95% CI 0.09–0.90).

### Number of events treated with antibiotics

Over the four-year period, the overall majority of patients reported that ‘none’ (41.3%–66.2%) of their reported events of sore throat were treated with antibiotics. A summary of these results is given in Table 5.

**Table 5:** Number of events treated with antibiotics as reported by the patient

	Number (%)
2013–2014	
All	41 (15.1)
Almost all	24 (8.8)
Half	11 (4.0)
Less than half	16 (5.9)
None	180 (66.2)
2014–2015	
All	43 (16.0)
Almost all	20 (7.4)
Half	13 (4.8)
Less than half	15 (5.6)
None	178 (66.2)
2015–2016	
All	46 (17.1)
Almost all	28 (10.4)
Half	10 (3.7)
Less than half	22 (8.2)
None	163 (60.6)
2016–2017	
All	89 (32.8)
Almost all	34 (12.5)
Half	15 (5.5)
Less than half	21 (7.7)
None	112 (41.3)

Ordinal logistic regression found no evidence of an association with SIMD (*p* = 0.781), sex (*p* = 0.187) and smoking status (*p* = 0.633), but there was evidence of an association with age (*p* = 0.005). Here, an increase in age (years) was associated with increased odds of not treating events with antibiotics (OR 1.02, 95% CI 1.01–1.04).

### Number of participants prescribed antibiotics by a GP prior to admission

Of the 279 study participants, 270 (96.8%) responded to the question of whether they had been prescribed antibiotics by their GP. Of these, 136 (48.7%) said they had been prescribed antibiotics by their GP prior to admission, and 134 (48.0%) said that they had not.

Binary logistic regression techniques found there was evidence of an association with age (*p* = 0.012), but no evidence of an association with sex (*p* = 0.471), smoking status (*p* = 0.056) and SIMD (*p* = 0.842). Here, increasing age (years) was associated with an increased likelihood of not being prescribed antibiotics.

### Previous hospital admissions

#### Tonsillitis

Of the 279 study participants, 180 (64.5%) provided responses: 29 (16.1%) said they had previously been admitted to hospital for tonsillitis, and 151 (83.9%) said that they had not.

#### Quinsy

Of the 279 study participants, 177 (63.4%) responded: 20 (11.3%) said they had previously been admitted to hospital for quinsy and 157 (88.7%) said that they had not.

#### Previous discussion with GP about a tonsillectomy

Of the 279 study participants, 273 (97.8%) provided a response: 61 (22.3%) said they had discussed a tonsillectomy with their GP and 212 (77.7%) said that they had not.

### Doctor's diagnosis for current admission

A doctor's diagnosis was available for 263 (94.3%) of the 279 study participants. Of these diagnoses, 168 (63.9%) were for tonsillitis, 94 (35.7%) were for quinsy and 1 (0.4%) was for DNSI.

Multinomial regression modelling found there was evidence of an association with sex (*p* = 0.001) and age (*p* = 0.003), but no evidence of an association with smoking (*p* = 0.094) and SIMD (*p* = 0.579).

### Meeting SIGN guidelines for tonsillectomy

Information on meeting SIGN guidelines for tonsillectomy was available for 262 (93.9%) of the 279 study participants. Of these, 195 (74.4%) did not meet the SIGN guidelines and 67 (25.6%) did.

Of the 67 study participants who did meet the SIGN guidelines for tonsillectomy, 64 had information related to a tonsillectomy discussion at the current visit. Of these, 20 (31.2%) were listed for tonsillectomy.

## Discussion

To our knowledge this is the first audit to assess throat morbidity in secondary care admissions with sore throat. This prospective audit has highlighted some key findings.

The mean age of 30.1 years reflected a similar large-scale UK audit of 1,725 patients presenting in the community (median 29.2, range 6–89).^11^ A similar audit in a Malaysian emergency department cohort of patients with low symptom scores had a mean age of 32.54 years.^12^

We found that fewer than 50% of patients admitted to the ENT department for a sore throat had been prescribed antibiotics prior to their admission. It is difficult to know exactly why this figure was so low. It could be that access to GP appointments has led to patients finding it difficult to get a primary care review when they have a sore throat. Furthermore, the NHS website for sore throat advice clearly states “Sore throats are very common and usually nothing to worry about. They normally get better by themselves within a week. You do not normally need antibiotics for a sore throat because they will not usually relieve your symptoms or speed up your recovery. You'll only be given antibiotics by a pharmacist or GP if you could have a bacterial infection”.^13^ Therefore, the public health message to patients is you do not normally need antibiotics, which may in part explain why only 50% of patients had been prescribed antibiotics prior to their admission; patients do not seek primary care review because they do not think they need antibiotics. Furthermore, GPs may be more likely to consider an alternative aetiology to bacterial tonsillitis, such as viral. It may also be that patients may present straight to their emergency department when in pain with a sore throat because no appointment is required and therefore it may be easier for the patient. Analysis of prescribing in 706 British family practices between 2002 and 2017 for all indications peaked in 2009 and have decreased since, although tonsillitis and peritonsillar abscess rates have risen once more within the same period, suggesting that there may be a relationship between primary care prescribing and hospital admission.^14^ It is difficult to know whether higher rates of antibiotic prescriptions by primary care could have resulted in fewer admissions. An audit of patients presenting in Nottingham with acute tonsillitis, peritonsillar cellulitis or quinsy showed that 58% had antibiotics prescribed in inadequate doses or even an inappropriate agent.^15^ There has been a fourfold increase in admissions in Scotland with DNSIs between 1996 and 2015.^16^ This is matched by an increased admission rate for tonsillitis and DNSIs in England of 310% and 39%, respectively, and a threefold rise in hospital admissions for tonsillitis in Wales of during the same period.^6,17^ This may also highlight the continued prevalence of patients attending the emergency department in the first instance with sore throat, rather than attending their GP.^18^ We also found that older patients were less likely to have been prescribed antibiotics prior to admission. It is unlikely that these patients were any less unwell or symptomatic because they were admitted. It is not clear why they were less likely to have had antibiotics before presentation, but it could be that GPs were more likely to consider an alternative aetiology to bacterial tonsillitis, or perhaps older patients did not go to their GP in the first place. Public health interventions have sought to minimise the number of patients requesting antibiotics for a variety of pathologies.^19^ The NHS has set up NHS Inform to allow the public to find accurate and reliable information on common medical conditions. NHS Inform advises that antibiotics are rarely necessary for sore throat and can increase side effects. It could be that the older population are more likely to follow official guidance. However, potential inadequate prescribing in the community must be correlated with diagnoses being made on clinical presentation. The Centor criteria were developed in 1982 to utilise the high sensitivity of symptoms and clinical signs in predicting Group A streptococcal infection, and the National Institute for Health and Care Excellence has since obviated the need for using rapid antigen testing of swabs for patients presenting with this problem in patients with a Centor score of 2–3 as having a limited effect on prescribing and outcomes.^20,21^ The more complex FeverPAIN score similarly stratifies likelihood of isolation of streptococcal infection purely on history, sufficient to de-escalate or delay antibiotics in 29% of cases.^22^ Therefore GPs have not routinely used point of care testing for sore throat in the primary care setting – clinical scoring with FeverPAIN or Centor are the standard method of assessing if antibiotics are required, despite both scores having poor overall diagnostic accuracy.^23^ For quinsy, the Liverpool Peritonsillar Abscess score has quoted sensitivity of 98% and a negative predictive value of 99% when using its five criteria, giving yet more scope to decrease prescribing antimicrobials without the need for further investigations.^24^ Furthermore, antimicrobial prophylaxis has shown to be ineffective in reducing the incidence of subsequent Group A streptococcus pharyngitis despite high antimicrobial susceptibility.^25–27^ One British cohort study has estimated as many as 1,000 antibiotic prescriptions may be required to prevent one episode of peritonsillar abscess.^28^ What this highlights clearly is that the diagnosis of tonsillitis and quinsy is still very challenging for primary care, with the decision making around antibiotic prescribing an ongoing problem. The pressures of the recent Group A streptococcus outbreak only compound this. Point of care testing is not standard practice in the UK; however, more research into this area should be considered.

This prospective audit has highlighted that patients admitted to hospital with sore throat have low-level, but long-term previous throat morbidity: as reported by patient recall, 25% of patients meet the SIGN guidelines for tonsillectomy when admitted. Responses on previous admission showed that 16.1% had been admitted before for tonsillitis and 11.3% had been admitted for quinsy. However, across the periods audited, 15%–27% (mean 20.1%) reported having between two and four overall tonsillitis events in preceding years, but a much lower percentage were reported to the GP. This represents a low burden of patients who seem to be having frequent episodes that are nearing the ‘7,5,3’ SIGN rule for tonsillectomy. Although very few patients had high numbers per year, the low number for years supports the finding of Douglas *et al* who demonstrated that patients on average had 27 episodes of tonsillitis over 7 years before achieving tonsillectomy. With the recent publication of the NATTINA trial, and more evidence being published about the long-term low-level tonsillitis people experience before achieving tonsillectomy, there is a strong argument for research into the benefit of lowering the SIGN guidelines. It also highlights that patients do not always seek medical advice when they have got tonsillitis, because it is often self-limiting. This may then mean that if episodes are not recorded by primary care, referral for consideration of tonsillectomy by primary care is delayed because of lack of well-documented episodes and patients ultimately suffer longer as a result. Innovative solutions such as an NHS Digital Sore Throat app for documentation of episodes may help improve this. We are also unsure why only 31% of patients in this study who met the criteria for tonsillectomy were listed for procedure. However, several explanations exist, such as on-call doctors being unaware of the SIGN criteria, or that certain departments may see patients back in clinic to be consented. We did not collect data on specific motives for being or not being added to the waiting list, so this low figure remains unexplained, but is an area that requires attention as ultimately if a patient fulfils the SIGN guidelines for tonsillectomy and wishes tonsillectomy they should be listed for tonsillectomy. The mean number of episodes of quinsy reported was 2.31, exceeding the 2 episodes considered best practice to complete tonsillectomy, implying that a more aggressive approach to tonsillectomy for this indication may be required.^29^

### Study limitations

The largest challenge to interpreting these data is the reliance on self-reporting of variables. Accuracy relies on correct recall, understanding of relative medical terms and respondent specificity. The vast majority of patient-reported episodes were found to be towards the milder end of the illness spectrum: 0–1 event and ‘none’ categories. Owing to the way in which the data were recorded (categories rather than specific numbers) we are unable to carry out certain statistical tests, which is a limitation of this study.

## Conclusions

This audit highlights the increasing burden of sore throat admissions to secondary care. Low-level, long-term tonsillitis was prevalent among this group, but only 25% met SIGN criteria for tonsillectomy. Many of the episodes of sore throat reported by patients were not reported to primary care, highlighting the need for innovative methods of recording tonsillitis episodes by the patient to ensure appropriate assessment of tonsillitis morbidity when patients are being considered/referred for tonsillectomy. The results, along with the recent high-profile NATTINA publication, suggest that there is an urgent need to reassess the ‘7,5,3’ rule, and that patients may benefit from a reduction in the number of sore throats needed per year to qualify for a tonsillectomy. We also suggest it would be both appropriate and efficient to offer elective tonsillectomy to all patients who on admission meet the SIGN criteria for tonsillectomy.
